# Supplementary material for: Asymmetric Synthesis of Quaternary Hydantoins via a Palladium-Catalyzed Aza-Heck Cyclization
Source: J Am Chem Soc. 2025 Nov 14;147(49):44692–8. doi: 10.1021/jacs.5c16022 (PMC12703750; doi:10.1021/jacs.5c16022)
Supplement: Supplementary file 2 [file ja5c16022_si_002.zip › All NMR FID Files/S32/S32_CarbonNMR/1/pdata/1/email_EM04-150-13C-morescans_1_1.pdf]

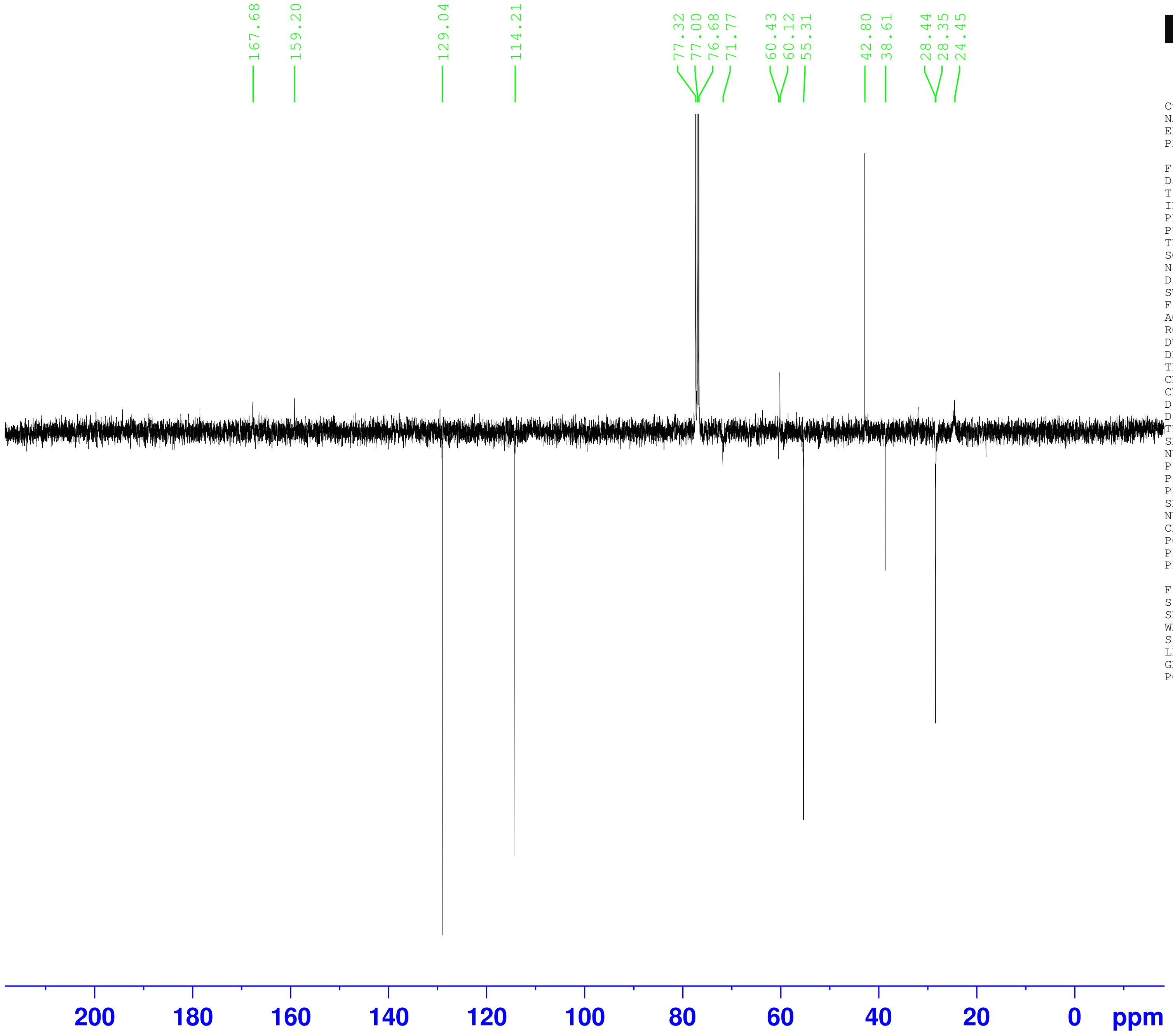

BRUKER

Current Data Parameters
NAME EM04-150-13C-morescans
EXPNO 1
PROCNO 1

F2 - Acquisition Parameters

Date_	20230929	
Time	2.36	h
INSTRUM	Avance Neo 400	U of DE Brown Lab
PROBHD	Z104450_0412 (	
PULPROG	jmod	
TD	65536	
SOLVENT	CDCl3	
NS	2048	
DS	4	
SWH	23809.524	Hz
FIDRES	0.726609	Hz
AQ	1.3762560	sec
RG	101	
DW	21.000	usec
DE	6.50	usec
TE	301.4	K
CNST2	145.0000000	
CNST11	1.0000000	
D1	5.00000000	sec
D20	0.00689655	sec
TD0	1	
SFO1	100.6228298	MHz
NUC1	13C	
P1	10.00	usec
P2	20.00	usec
PLW1	44.93500137	W
SFO2	400.1316005	MHz
NUC2	1H	
CPDPRG[2	waltz65	
PCPD2	90.00	usec
PLW2	8.31639957	W
PLW12	0.23101000	W

F2 - Processing parameters

SI	32768	
SF	100.6127685	MHz
WDW	EM	
SSB	0	
LB	1.00	Hz
GB	0	
PC	1.40	
